# The Role of Hyaluronic Acid in the Treatment of Gingivitis and Periodontitis at Different Stages: A Systematic Review and Meta-Analysis with Short-Term Follow-Up

**DOI:** 10.3390/bioengineering12111135

**Published:** 2025-10-22

**Authors:** Nansi López-Valverde, Norberto Quispe-López, Javier Flores Fraile, Antonio López-Valverde, Bruno Macedo de Sousa, José Antonio Blanco Rueda

**Affiliations:** 1 Department of Surgery, Instituto de Investigación Biomédica de Salamanca (IBSAL), University of Salamanca, 37007 Salamanca, Spain; nlovalher@usal.es (N.L.-V.); norberto_quispe@usal.es (N.Q.-L.); j.flores@usal.es (J.F.F.); jablancor@usal.es (J.A.B.R.); 2 Institute for Occlusion and Orofacial Pain, Faculty of Medicine, University of Coimbra, Polo I-Edifício Central Rua Larga, 3004-504 Coimbra, Portugal; bsousa@fmed.uc.pt

**Keywords:** hyaluronic acid, gingivitis, periodontitis, periodontitis therapy, randomized controlled trials, systematic review, meta-analysis

## Abstract

Periodontal diseases are inflammatory conditions that destroy the periodontal attachment apparatus. Hyaluronic acid (HA) has anti-inflammatory properties that make it a candidate for the adjuvant treatment of gingivitis and periodontitis. Our objective was to observe the role of HA in the variability of clinical parameters indicative of gingivitis/periodontitis by comparing it with conventional treatments or placebo. This systematic review and meta-analysis was conducted according to Cochrane guidelines, and searches were performed in PubMed, Embase, Cochrane Central, Scopus, and Web of Science (WOS) to identify eligible studies. Review Manager 5.4.1 and SPSS Statistics 30.0^®^ were used to calculate standardized mean differences (SMDs) and 95% confidence intervals (CIs). The outcomes assessed were probing depth (PPD), bleeding on probing (BOP), clinical attachment level (CAL), plaque index (PI), and gingival index (GI). Sixteen randomized clinical trials (RCTs) with 947 subjects were included. HA as an adjunct to periodontal treatment improves the clinical parameters of PPD in the short and medium term (1–24 months, 12.5 average) (−0.51; 95% CI [−0.85 to −0.17]; *p* = 0.004), BOP, CAL and GI. Plaque indices (PI) approached statistical significance. Despite limitations and heterogeneity, the evidence reveals that only two of the included studies on severe periodontitis reported significant improvements in CAL gain and PPD reduction, with attachmet gains greater than 1 mm at 12 months of follow-up.

## 1. Introduction

Hyaluronic acid (also known as hyaluronan, HA) is a polysaccharide of the non-sulfated acid glusaminoglycan type, and it is one of the most abundant molecules in the extracellular matrix (ECM) of many tissues, including periodontal soft (gingiva and periodontal ligament) and hard (alveolar bone and cementum) tissues [1]. It is considered that it may play a role in regulating the inflammatory response through its degradation into low-molecular-weight molecules in chronically inflamed tissues [2].

Most cells in the human organism synthesize HA, so it plays a key role in different biological processes that confirm its potential as a therapeutic option in several pathologies [3].

It has been reported that different molecular sizes of HA have different biological activities. High-molecular-weight HA suppresses the immune response, preventing excessive increases in inflammation. Low-molecular-weight HA promotes angiogenesis and tissue remodeling in the wound healing process, particularly in gingival tissue in the early stages of periodontitis, possibly due to the action of hyaluronidase [4,5]. Several in vitro studies have demonstrated the ability of HA to increase proliferation and early osteogenic differentiation [6], and recent reviews have highlighted its usefulness in healing processes and tissue regeneration [7,8]. Other properties that support its use are its biocompatibility; it is well tolerated and does not cause immune responses or adverse reactions. As it is not immunogenic, it does not stimulate an immune response or cause inflammation [9]. In recent years, some studies have attributed antibacterial properties to it [10,11]. The ability of HA to inhibit microbial aggression during the healing process appears to be related to its ability to reduce bacterial adhesion. This bactericidal capacity would prevent or delay the growth of bacteria without destroying them [12,13].

Periodontal diseases are a group of pathologies of inflammatory origin, which cause loss of attachment and destruction of the alveolar bone, leading, if left untreated, to tooth loss [14]. It is precisely this particular aspect of chronic inflammatory disease that has led to the current use of HA in the treatment of these pathologies, mainly in relation to soft tissues [15].

In addition to its anti-inflammatory and bacteriostatic properties, it has even been attributed with certain osteoinductive properties, which contribute to the healing of xenografts by increasing the percentage of new bone formation and reducing the percentage of residual graft [16].

**Figure 1:**
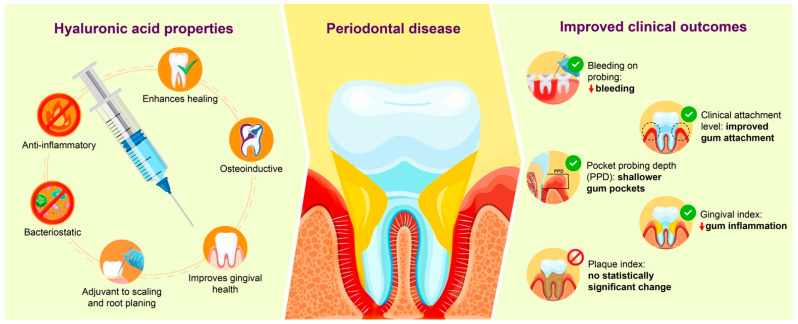
Graph showing the properties of HA.

In patients with gingivitis and periodontitis, HA has been used as an adjuvant therapy for scaling and root planning therapy (SRP), having demonstrated its ability to reduce prostaglandins, metalloproteinases and bioactive materials, which hinders tissue destruction, favoring healing [17]. Other studies have also demonstrated its usefulness in gingivitis via topical application, decreasing bleeding and improving gingival health [18,19] (Figure 1).

There are few systematic reviews [20,21,22,23,24], and updates are recommended every 2 to 5 years [25], so the aim of our study was to evaluate the clinical efficacy of HA in gingivitis and periodontitis (Stages II, III, IV, and chronic periodontitis) through a systematic review and meta-analysis.

## 2. Materials and Methods

### 2.1. Protocol and Registration

This systematic review and meta-analysis were carried out in accordance with the guidelines of the Cochrane Handbook [26]. The protocol of this meta-analysis was registered in PROSPERO ID: CRD42024626469 on 22 December 2024.

### 2.2. Question of Interest and PICO Format

The research question was formulated according to the PICO format: Is HA treatment effective as a sole or adjunctive therapy for the treatment of gingivitis/periodontitis in adult patients?

Interventional studies in adult patients with gingivitis, periodontitis or both (P) comparing HA treatment (I) with patients receiving conventional treatment or no treatment (C) were included to observe increase/decrease effects on clinical parameters (O), with only randomized clinical studies considered (Table 1).

### 2.3. Study Inclusion and Exclusion Criteria

The research studies were selected according to the following inclusion criteria: (i) randomized clinical trials (single or double blind) that included more than 10 adult subjects (≥18 years of age); (ii) that included subjects suffering from gingivitis or periodontitis; (iii) that provided data on clinical parameters indicative of these pathologies; (iv) with statistical methods that included means and standard deviation, together with units with which to quantify mediator levels; (v) that were published in English. Studies that did not follow all the criteria defined above, with lack of data demonstrating periodontal disease, in vitro or experimental animal studies, case series or clinical cases, literature reviews and irrelevant studies, such as editorials, contributions to congresses, etc., were excluded.

### 2.4. Search Strategies

Two reviewers (NL-V, AL-V) independently searched PubMed via Medline, Embase, Scopus and Cochrane Central databases and the Web of Science (WOS) scientific information service until 30 June 2024, using Medical Subject Headings (MeSH) terms and keywords:

“hyaluronic acid” [MeSH Terms]; “hyaluronic” [All Fields]; “acid” [All Fields]; “Humans” [MeSH terms]; “gingivitis” [MeSH Terms]; “periodontitis” [All Fields]; “periodontal diseases” [MeSH Terms] for PubMed-Medline.

“gingivitis” [Title/Abstract]; “periodontitis” [Title/Abstract]; “hyaluronic acid” [Title/Abstract]; “gingivitis” [MeSH Terms]; “periodontitis”; “periodontal diseases” [MeSH Terms]; “hyaluronic acid” [MeSH Terms] for Embase.

“hyaluronic acid”; “gingivitis”; “periodontitis”; “periodontal diseases”; “Humans” for Cochrane Central.

“hyaluronan”; “hyaluronic acid”; “gingivitis”; “periodontitis”; “periodontal diseases”; “Humans” for WOS.

“hyaluronic acid”; “gingivitis treatment”; “periodontitis treatment” for Scopus.

In addition, a manual search was conducted, and grey literature (Teseo, SciELO, ProQuest and Google Scholar, focusing on this search engine in the first 250 results) was consulted; the bibliographic references of the selected studies were also consulted to obtain as much information as possible. No restrictions were applied in terms of geographical location or time periods (Table 2).

### 2.5. Data Collection

Two reviewers (NL-V and AL-V) extracted and tabulated the data from each included study using the standardized data extraction tool “The Joanna Briggs Institute Meta-Analysis of Statistics Assessment and Review Instrument” (JBI-MAStARI) [27]. The two reviewers reviewed the titles and abstracts of the pre-selected studies. Those that met the inclusion criteria were read in full and the data extracted. Disagreements between the reviewers were resolved through discussion and mediation by a third reviewer (JABR). Cohen’s kappa (κ) index [28] was used to assess inter-rater agreement. The data extracted from the studies included specific details of the interventions, study methods, populations, specific objectives and significant results to formulate the question of interest. The results were entered twice to minimize error bias.

### 2.6. Statistical Analysis

For the analysis and synthesis of data obtained from the selection of RCTs, Review Manager software (RevMan Software. Version 5.4.1; The Cochrane Collaboration, Copenhagen, Denmark; 2020) and IBM SPSS Statistics V.30^®^ software were used. The effectiveness of HA in gingivitis and periodontitis was evaluated by means of a meta-analysis for each of the clinical parameters analyzed. All were based on the standardized mean difference (SMD) and the confidence interval (95% CI). Heterogeneity was considered unimportant with I^2^ = 0–30%; moderate, I^2^ = 40–50%; substantial I^2^ = 60–75% and considerable I^2^ ≥ 75% [29]. The threshold for statistical significance was set at *p* < 0.05. Due to the heterogeneity of the results, a random effects meta-analysis was performed.

### 2.7. Risk of Bias and GRADE Assessment

Two investigators (NL-V and AL-V) independently assessed the risk of bias of studies using the Cochrane Risk of Bias Tool (RoB2, the 22 August 2019 version) [30], using 7 domains: Random sequence generation (Selection bias); Allocation concealment (Selection bias); Blinding of participants and personnel (Execution bias); Blinding of outcome assessment (Detection bias); Incomplete outcome data (Attrition bias); Selective reporting (Reporting bias); Other bias. The certainty of the evidence was assessed using the GRADE approach for each meta-analysis [31] as “high,” “moderate,” “low,” or “very low.” Since all the individual studies we analyzed were RCTs, the initial assessment was designated as “high,” which could then be downgraded to “moderate” (1 step), ‘low’ (2 steps), or “very low” (3 steps) based on various factors that reduce the certainty of the evidence, such as a high risk of bias. However, downgrading the certainty may be offset by other factors that may increase certainty, such as a large effect size and a dose–response gradient. Discrepancies among the evaluators were discussed to reach consensus.

## 3. Results

**Figure 2:**
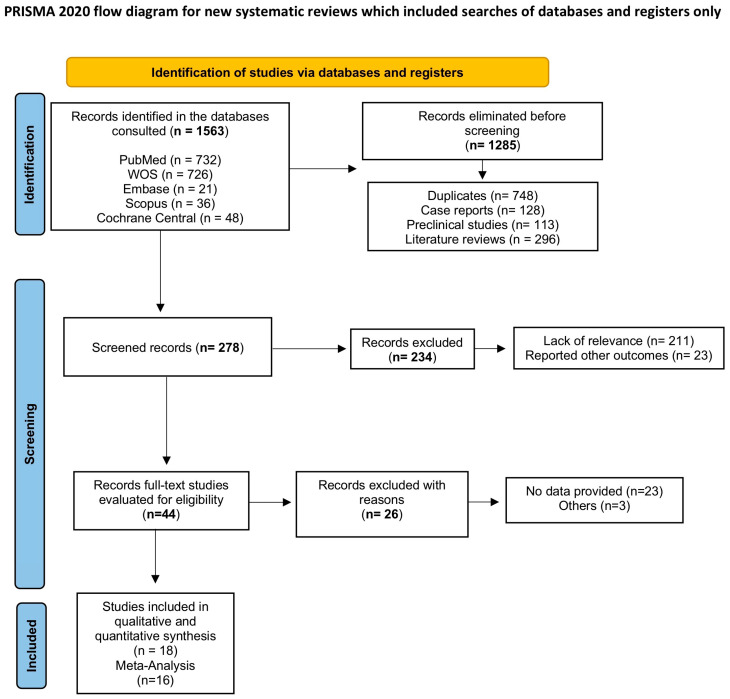
Flowchart.

Figure 2 (Flowchart) shows the results obtained in the different stages of the literature search. Of the 1563 records identified, 1285 were eliminated (duplicates, case reports, preclinical studies, literature reviews); subsequently, 234 were eliminated (text not accessible, lack of relevance, and communication of other results); finally, of the 44 studies selected for evaluation, 26 were eliminated for various reasons, leaving 18 studies for the systematic review [31,32,33,34,35,36,37,38,39,40,41,42,43,44,45,46,47,48] and only 16 studies for meta-analysis [32,33,35,36,37,38,39,40,41,42,43,44,46,47,48,49]. The agreement between reviewers when including studies exceeded 85% (κ > 85%).

### 3.1. Characteristics of the Studies

A total of 947 subjects were studied, and the mean age ranged from 18 to 78 years. Two of the included studies [37,43] were the most complete, as they investigated all 5 clinical parameters considered in our meta-analysis (PPD; BOP; CAL; PI; GI); 8 studies [32,35,40,42,46,47,48,49] investigated 4 of the parameters (PPD; BOP; CAL; PI); 4 studies [36,37,41,43] investigated 3 parameters (PPD, CAL, BOP); 1 study [33] investigated 2 parameters (PPD, CAL); and only 1 of the studies selected for meta-analysis [34] investigated the effect of HA on gingivitis. The follow-up periods ranged from 1 to 12 months, the most common follow-up being 6 months. Studies with different follow-up periods were included primarily to increase statistical power. Only three studies [33,35,42] provided results for CAL and PPD in stage III and IV periodontitis after 12 months; the study by Bertl et al. [35] only reported results for PPD. Among the studies analyzed, Olszewska-Czyz et al. [43] presented the largest sample with 100 patients. All the studies included non-smoking subjects, except the studies by Devina et al. [37], Mohammad et al. [38], Mallikarjun et al. [46] and Johannsen et al. [49], which included in their respective studies subjects who smoked in their different forms (tobacco or snuff). Only 6 studies out of the 18 included [32,33,35,36,37,41] registered their research on the ClinicalTrials.gov platform. Although the most commonly used statistical tests were Shapiro–Wilk and Wilcoxon, the study by Mohammad et al. [38] did not clearly indicate the statistical tests used in their research. Several studies [39,41,42,43,45,46,48,49] did not consider reporting adverse effects, despite the relevance of this aspect in research, and only 2 studies [34,38] reported them in their respective investigations. The journal most frequently used to publish the studies was Clinical Oral Investigations [35,36,39,41,42,48].

The general and specific characteristics of the included studies are shown in Table 3 (Stages II, III, IV periodontitis). Table 4 shows the results for CAL and PPD (stages III and IV) at 12 months of follow-up. Table 5 shows the specific and sociodemographic characteristics of the included studies.

### 3.2. Overall Meta-Analysis

Only 16 studies were used for the meta-analysis; the study by Axe et al. [34] was not used because it was the only one evaluating the effect of HA on gingivitis and, as such, was not comparable, and the study by Al-Shammari et al. [45] was not used because it provided the data in the form of graphs. Individual meta-analyses were performed for each of the clinical parameters considered (PPD, CAL, BOP, PI, GI). In all cases, heterogeneity was considerable (I^2^ ≥ 75%), ranging from 79 to 90%. Given the high heterogeneity of the studies, a sensitivity analysis was performed by excluding studies, which revealed that the studies by Devina et al., Mohammad et al., and Pilloni et al. [37,38,40] were responsible for the high heterogeneity. One subgroup analysis was performed for studies comparing the intervention group (HA) with SRP alone, and another for studies comparing HA with placebo, as the other studies (toothpaste, etc.) were not comparable due to their scarcity. No analysis of adverse effects was performed, due to lack of data.

#### 3.2.1. Meta-Analysis for PPD Parameter

**Figure 3:**
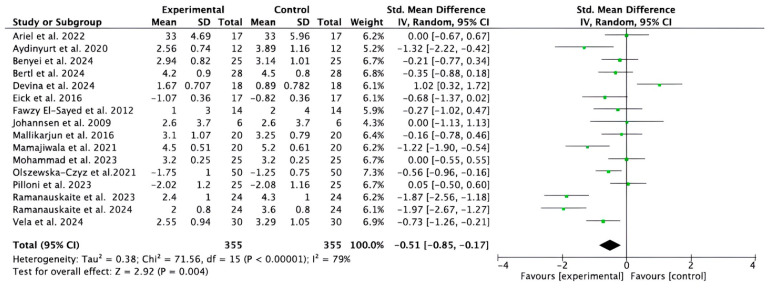
Forest plot of the overall reduction in the PPD in the short and medium term. CI, Confidence Interval; SD, Standard Deviation [32,33,35,36,37,38,39,40,41,42,43,44,46,47,48,49].

Sixteen studies [32,33,35,36,37,38,39,40,41,42,43,44,46,47,48,49] provided data on the effects of HA as an adjuvant in the reduction of PPD. High heterogeneity was identified in the included studies (I^2^ = 79%). The meta-analysis showed a significant reduction in PPD in the short and medium term (from 2 weeks to 12 months) in the experimental groups compared to the controls (−0.51; 95% CI [−0.85 to −0.17]; *p* = 0.004) (Figure 3). The study reports indicate that complementary treatment with HA produced significant results in terms of PPD, compared with the control groups, with values ranging from *p* < 0.05 to *p* ≤ 0.0001.

#### 3.2.2. Meta-Analysis for CAL Parameter

**Figure 4:**
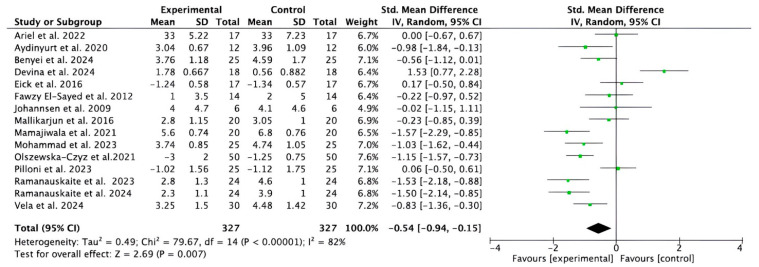
Forest plot of the gain in CAL in the short and medium term. CI, Confidence Interval; SD, Standard Deviation. Statistical significance *p* = 0.007 [32,33,36,37,38,39,40,41,42,43,44,45,46,47,48,49].

Fifteen studies [32,33,36,37,38,39,40,41,42,43,44,45,46,47,48,49] provided data on short- and medium-term CAL gain. Despite considerable heterogeneity (I^2^ = 82%), the meta-analysis showed that adjuvant HA significantly improved CAL compared to control treatments (−0.54; 95% CI [−0.94 to −0.15]; *p* = 0.007) (Figure 4). The studies analyzed reported that complementary treatment with the study reports indicate that HA produced statistically significant results compared to the control groups in terms of CAL gain, with values ranging from *p* < 0.001 to *p* < 0.0001.

#### 3.2.3. Meta-Analysis for BOP Parameter

**Figure 5:**
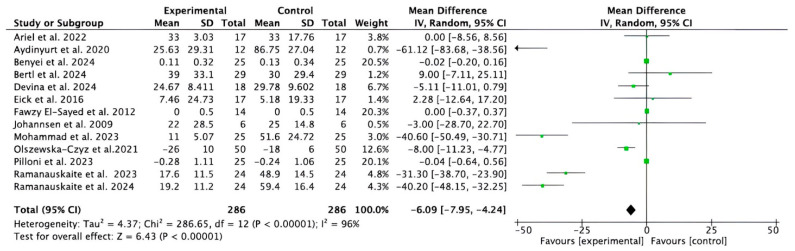
Forest plot of the overall reduction in the BOP in the short and medium term. CI, Confidence Interval; SD, Standard Deviation [32,35,36,37,38,39,40,41,43,44,47,48,49].

Thirteen studies [32,35,36,37,38,39,40,41,43,44,47,48,49] provided data for short- and medium-term BOP. The meta-analysis (despite the considerable heterogeneity of the studies) showed that adjuvant HA significantly improved BOP compared to control treatments (−6.09, 95% CI [−7.95 to −4.24]; *p* < 0.00001; I^2^ = 96%) (Figure 5).

#### 3.2.4. Meta-Analysis for GI Parameter

**Figure 6:**
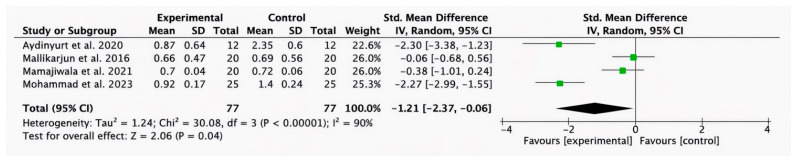
Forest plot of the overall short-term GI reduction. CI, Confidence Interval; SD, Standard Deviation [38,42,44,46].

Four studies [38,42,44,46] provided data for short-term GI. The meta-analysis showed that HA, compared to conventional treatment, improves GI rates (−1.21, 95% CI [−2.37 to −0.06]; *p* = 0.04; I^2^ = 90%) (Figure 6).

#### 3.2.5. Meta-Analysis for PI Parameter

**Figure 7:**
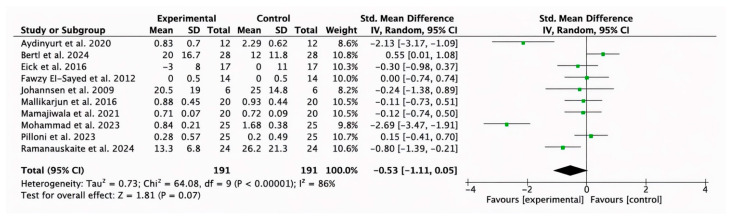
Forest plot of the overall reduction in the PI in the short and medium term. CI, Confidence Interval; SD, Standard Deviation [32,35,38,40,42,44,46,47,48,49].

Ten studies [32,35,38,40,42,44,46,47,48,49] reported the short- and medium-term results of PI. The meta-analysis showed a trend towards statistical significance between the experimental and control groups (−0.53, 95% CI [−1.11 to 0.05]; *p* = 0.07; I^2^ = 86%) (Figure 7).

In all the parameters studied, the sensitivity analysis showed that the elimination of any study did not change the results.

#### 3.2.6. Subgroup Analysis for HA (Test) Compared with SRP Alone

**Figure 8:**
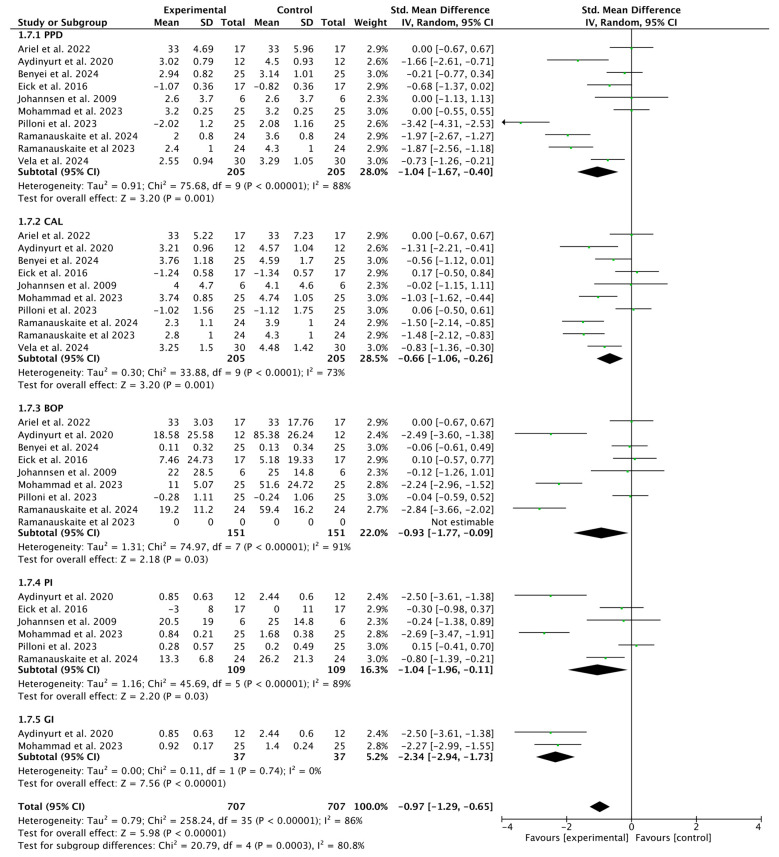
Forest plot of the HA Test subgroup and SRP alone (control) [32,33,36,38,39,40,41,44,47,49].

The results suggest that the experimental intervention has a positive and statistically significant effect on improving periodontal parameters (PPD, CAL, BOP, and PI), but the high heterogeneity (I^2^ between 73% and 91%) limits confidence in the actual magnitude of the effect (−0.97, 95% CI [−1.29 to −0.65]; *p* = 0.0003 (Figure 8).

#### 3.2.7. Subgroup Analysis of HA (Test) Compared with Placebo

**Figure 9:**
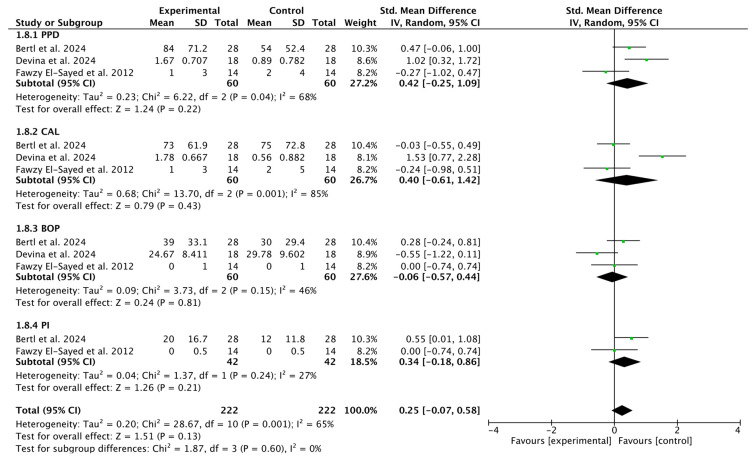
Forest plot of the HA and placebo subgroup [35,37,48].

Overall, the *p*-value > 0.05 in all results confirms that there are no statistically significant differences between the interventions. However, the I^2^ values show that consistency between studies varies considerably, especially in PPD and CAL (0.25, 95% CI [−0.07 to 0.58]; *p* = 0.60; I^2^ = 0%) (Figure 9).

### 3.3. Risk of Bias

Risk of bias assessment is one of the pillars of evidence-based medicine, so two re-viewers (NL-V and AL-V) independently analyzed the quality of included studies according to the Cochrane Risk of Bias tool [31]. Disagreements between the two were resolved by discussion. RCTs were assessed in 7 domains: the randomization process; deviations from intended interventions; sparse or missing outcome data; measurement of those outcomes; and selection of the reported outcome; a final bias that is related to the other biases. According to the Cochrane Handbook for Systematic Reviews of Interventions, a “high” rating was given to studies considered to be at high risk of bias, “low” to those at low risk of bias, and “borderline” to those with uncertain bias or lack of information on potential bias. However, some studies included randomization software, and it was difficult for the assessors to know which domains they addressed and which they did not. The domains “blinding of outcome assessment” (detection bias) and “blinding of participants and staff” (performance bias) had the highest uncertainty, and the study by Johannsen et al. [48] had the highest risk, especially for uncertainty in domains 2, 3, and 4. Overall, 57% of studies had a low risk of bias, 37% had a moderate risk, and only 6% had a high risk of bias.

**Figure 10:**
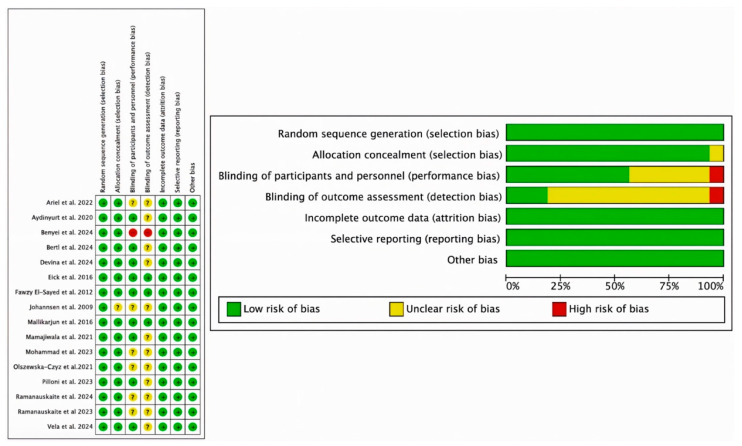
Representative graph of the bias stiffness of the studies included in the meta-analysis, assessed by means of the tool (RoB2) [32,33,35,36,37,38,39,40,41,42,43,44,46,47,48,49].

Nevertheless, the included studies met most of the domains and were considered to have a moderate–low risk of bias (Figure 10).

### 3.4. Publication Bias

**Figure 11:**
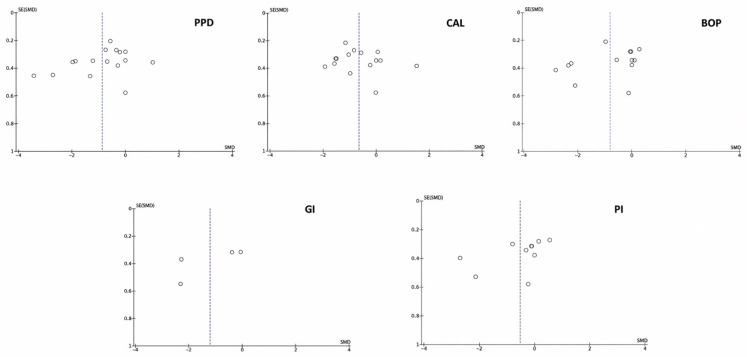
Funnel plots of publication bias in the studies analyzed.

The graph in Figure 11 represents the results of the publication bias. The *x*-axis shows the results of the studies, and the *y*-axis represents the accuracy of the studies. The observed asymmetry demonstrates a considerable publication bias.

## 4. Discussion

To the best of our knowledge, our systematic review with meta-analysis is the first in recent years to focus on evaluating the role of HA as an adjunct in the treatment of periodontal disease. Although 18 studies were identified that evaluated its ability, only 16 were useful for meta-analysis. The quantitative synthesis of the studies that compiled sufficient data to carry out a meta-analysis showed that the adjuvant use of HA in the treatment of periodontitis provides clinical advantages in comparison with conventional treatment or other types of treatment. Our meta-analysis showed a high level of heterogeneity (between 79 and 90%), possibly due to differences in the severity of the disease (degree of periodontitis), the characteristics of the population, the formulations and application of HA, as well as the location of the defects. All this makes it difficult to properly evaluate the product in question. Furthermore, the follow-up times and the different statistical programs used in the different studies can lead to biases in the communication and interpretation of the results.

Periodontal diseases (plaque-induced gingivitis and periodontitis) are chronic inflammatory diseases that affect the tissues that support the teeth, and several studies support the use of HA as an adjuvant therapy in their treatment [50,51,52,53]. Our meta-analysis, in which HA was used as a complementary treatment in periodontal therapy, achieved statistical significance for the parameters PPD (*p* = 0.0003), CAL (*p* = 0.007), BOP (*p* = 0.0003) and GI (*p* = 0.04), and showed a trend towards statistical significance in PI (*p* = 0.07). In general, in patients with stage III/IV periodontitis, complementary treatment with HA produced statistically significant results for CAL gain [32,33,39,41,42], with values ranging from *p* < 0.001 to *p* < 0.0001. These results are in line with those of other previous reviews [17,54]. However, it should be noted that only two of the included studies [33,42] reported significant results for this clinical parameter, with insertion gains greater than 1 mm at 12 months of follow-up. The study by Pilloni et al. [40], on residual pockets, reported similar results between the intervention and control groups.

It was also demonstrated that the addition of HA to conventional therapy produced a reduction in bleeding on probing (−6% with respect to the control group) and an increase in periodontal attachment (1 mm more than the control group) [43]. Benyei et al. and Ramanauskaite et al. [36,39] found statistically significant differences in PPD, CAL and BOP (*p* < 0.001) at 6 and 9 months, respectively, with the use of high-molecular-weight HA in the first study and cross-linked HA in the second, after subgingival instrumentation. In view of these results, it appears that the use of HA as an adjunct to periodontal therapy would improve parameters of vital importance in periodontal disease, such as CAL and PPD, only in advanced stages of the disease, with BOP being the periodontal index that obtains the highest reductions, although its clinical relevance would not be equivalent to that of CAL and PPD.

There are two publications from the same study [32,39]: the 2023 publication focuses on gingival and periodontal parameters, while the study published in 2024 focuses on microbiological parameters, although it also reports clinical outcomes (PPD, CAL, BOP, PI). Despite obtaining robust results that increase the statistical power of our meta-analysis, we wish to highlight our concern regarding the independence of the two studies. Their results are in line with those found by Diehl et al. [52] in a retrospective study in which subgingival cross-linked HA was used to treat persistent deep periodontal pockets, observing clinically relevant improvements in PPD reduction, CAL gain and BOP frequency. These results are consistent with those obtained by Iorio-Siciliano et al. [54], who also found clinical benefits when combining non-surgical periodontal treatment and the use of HA in infraosseous defects. These findings confirm the regenerative, anti-inflammatory, and healing properties of HA, which are highly relevant in severe cases of periodontitis, where conventional treatments may be insufficient [55]. However, a consensus report from the American Academy of Periodontology considers that surgical intervention remains the treatment of choice for intraosseous defects [56].

For several years now, microsurgical techniques (MIST) and modified microsurgical techniques (M-MIST), either alone or in combination with biomaterials, have led to clinical improvements that are evident in reduced probing depth and increased clinical attachment [57]. However, none of the included studies compared the complementary use of HA with microsurgical techniques. We also found no references in the literature on the combination of HA as a complement to these techniques, and further research is needed to assess whether HA can provide additional benefits when combined with these minimally invasive approaches.

HA formulations and concentrations varied in the different studies, with the latter ranging from relatively low concentrations (0.2 and 0.3%) [34,35,37,46] to higher concentrations [38,42,45,47]. These discrepancies prevent the authors of this review from recommending appropriate concentrations and formulations for the use of the product.

In this regard Mohammed et al. [58] tested different formulations of high-molecular-weight HA, crosslinked and non-crosslinked, in an in vitro model of oxidative stress-induced human oral mucosal injury and in an in vivo murine model of oral/intestinal mucositis, and found that all the formulations tested provided the same protection against oxidative stress-induced damage; all equally prevented apoptotic damage and reduced COX-2 enzyme activity.

Our meta-analysis included studies that used HA in different clinical settings, either as an adjunct to nonsurgical periodontal treatment [32,35,36,37,38,39,40,41,44,45] or surgical treatment [33,42,43,48], in cases of stage II, III, and IV periodontitis and chronic periodontitis, which could bias the results of the meta-analysis. However, the objective of both protocols was to surgically remove the entire lining of the pocket without any type of remodeling of the bone contour, before applying the HA gel in direct contact with the connective tissue (insertion epithelium), so that it could exert all its supposed beneficial effects, and this encouraged us to include the last three studies [59]. In fact, we recognized that only one of the studies included [45] strictly evaluated the effect of subgingival application of 0.8% HA gel as a complement to SRP in patients with moderate to severe periodontitis, clinically observing that all indices, except CAL, showed a statistically more significant reduction in the test sites than in the control sites at 6 and 12 weeks, results very much in line with those found by us.

On the other hand, two studies included in the meta-analysis [31,38] used sodium hypochlorite in combination with HA to improve their results. In this sense, Ioro-Siciliano et al., in a randomized study [60], drew attention to the antimicrobial effects of sodium hypochlorite and its use as an adjuvant in the treatment of periodontitis and Jurczyk et al. did so in an in vitro study [61]. Diehl et al. [52] demonstrated, through the association of HA and sodium hypochlorite, a reduction in PPD greater than 2 mm and a similar gain in CAL (2.02 mm) in a retrospective study with 29 patients and 111 sites treated. Bleeding on probing was reduced by more than 60% and pocket closure occurred in almost 25% of cases. It is surprising that one of the included studies resorted to the use of chlorhexidine as a postoperative rinse in the retreatment of residual pockets by subgingival instrumentation and a mixture of polynucleotides with HA [40]. The conclusions of the study were that such measures could improve the healing of periodontal wounds but were not relevant in comparison with subgingival reinstrumentation alone, results consistent with our meta-analysis, in which subgroup analysis comparing the intervention group (HA) with SRP alone showed a statistically significant effect on the improvement of periodontal parameters (*p* = 0.0003).

In this respect, it has been reported that clinically used chlorhexidine stops cell migration and reduces the survival of fibroblasts and osteoblasts in vitro [62,63] and that the adverse results of the study could be due to this negative effect of chlorhexidine on the survival of these cell species. On the other hand, a recent in vitro study [64] reported the greater antimicrobial potency of a modified HA gel compared to other products on the market (such as chlorhexidine), for the prevention and treatment of periodontal disease, without presenting side effects or lack of long-term tissue biocompatibility. Preclinical studies have demonstrated that intraosseous defects, gingival recessions, and furcation defects treated with HA gel showed a greater area of new cementum and new periodontal ligament [65,66]. These preclinical observations have been confirmed in clinical trials that found substantial benefit from the use of HA in gingival recessions and intraosseous defects [41,67].

In general, the complementary use of HA produces benefits in terms of reducing inflammatory reactions and changes in pocket depth, improving the level of clinical attachment, although the professional would have to evaluate the cost/benefit ratio with the adjuvant use of the product. The included studies did not present any potential risks or side effects following the use of HA as a complement to periodontal therapy, despite the fact that none of the sixteen studies considered in this meta-analysis presented maintenance programs with products that included HA in their formulation.

The use of HA in combination with other products, such as probiotics, photodynamic therapy and tetracycline fibers, which have proven effective in periodontal healing, should also be considered [68,69].

Finally, we should point out that some studies [33,34,35,40,41,47] received commercial funding to carry out their investigations.

Systematic reviews are, essentially, analyses of evidence and results (cause-effect) from the available scientific literature and make a judgment about the effectiveness of a treatment in one or more pathologies, which involves a series of complex steps that can inevitably lead to biases and limitations.

Our systematic review was limited to RCTs published in English, which could lead to bias; however, we only considered RCTs because they are the only studies suitable for a quality meta-analysis (qualitative and quantitative components). On the other hand, the most recent reviews on the effectiveness of HA in periodontal diseases are from 2022 and 2025 [21,22,23]; two others [20,24] are older. The review by Bhati et al. [21], despite including a series of studies, is a narrative review and, as such, does not quantify the effect of HA, limiting itself to a non-systematic search for studies, without answering a research question. The review by Karakostas et al. [22] is a more comprehensive review carried out according to PRISMA criteria (Table S1); however, it lacks a meta-analysis due to the small number of RCTs with low risk of bias. In addition, they focused exclusively on the treatment of gingivitis. Nevertheless, they concluded that the complementary use of HA improves the clinical course when combined with gingivitis treatment, whether surgical or non-surgical. The latest review by Inchingolo et al. [23], which coincides with ours, focuses on probing depth and clinical attachment level and, unlike our study, lacks a complementary meta-analysis.

## 5. Limitations and Future Directions

The studies included in our meta-analysis showed high publication bias, as well as considerable heterogeneity, ranging from 79% to 90%, which could be influenced by differences in disease severity (different stages), differences between the populations studied, the location of the defects, and the lack of standardized formulations. All of this makes it difficult to evaluate the product being tested. In addition, follow-up times and the different statistical programs used in the various studies may lead to biases in the communication and interpretation of results. The inclusion of publications exclusively in English and databases that were not consulted could also lead to publication bias.

Finally, it is worth mentioning one aspect, referring to the Hawthorne effect [70], which causes changes in the behavior of people who feel observed in epidemiological studies, something that was not taken into account in any of the studies included.

## 6. Conclusions

Despite the limitations of our study and the heterogeneity of the results, also taking into account linguistic bias, the current evidence supports the clinical benefits at twelve months of follow-up of subgingival HA application as an adjunctive treatment for periodontitis, particularly with regard to improving bleeding indices and pocket depth, as well as increasing clinical attachment levels only in advanced periodontitis cases with clinical relevance. Given these limitations, well-designed RCTs with long-term follow-ups and standardized HA formulations are needed to reinforce these conclusions.

## Figures and Tables

**Table 1:** PICO format.

Population	Adult subjects suffering from gingivitis or periodontitis
Intervention	HA, either as a single or adjuvant treatment
Comparisons	Conventional or placebo treatment
Outcomes	Observe the effects of treatment on clinical parameters indicative of gingivitis/periodontitis (Δ PPD; Δ BOP; Δ CAL; Δ PI; Δ GI)
Study design	RCTs

Δ, variable alteration; PPD, Pocket probing depth; BOP, Bleeding on probing; CAL, Clinical attachment level; PI, Plaque index; GI, Gingival index; RCTs, Randomized Controlled Trials.

**Table 2:** Search strategy. Search strings.

Databases	Search Details
PubMed via Medline	“hyaluronic acid” [MeSH Terms] OR “hyaluronic” [All Fields] AND “acid” [All Fields] AND “Humans” [MeSH terms]. “hyaluronic acid” [MeSH Terms] OR “hyaluronic” [All Fields] AND “acid” [All Fields] AND “gingivitis” [MeSH Terms] OR “periodontitis” [All Fields] OR “periodontal diseases” [MeSH Terms].
Embase	“gingivitis” OR “periodontitis” [Title/Abstract] AND “hyaluronic acid” [Title/Abstract]. “gingivitis” [MeSH Terms] OR “periodontitis” OR “periodontal diseases” [MeSH Terms] AND “hyaluronic acid” [MeSH Terms].
Cochrane Central	“hyaluronic acid” AND “gingivitis” OR “periodontitis” OR “periodontal diseases” AND “Humans”.
Web of Science	“hyaluronan” OR “hyaluronic acid” AND “gingivitis” OR “periodontitis” OR “periodontal diseases” AND “Humans”.
Scopus	“hyaluronic acid” AND “gingivitis treatment” OR “periodontitis treatment”
Boolean operators	AND and OR

**Table 3:** Characteristics of studies and participants included in the meta-analysis.

Study, Year	Type of Study	Subjects Number	Pathology	Diagnostic Criteria	HA Type and Concentration	Control Treatment	Periodontal Parameters Involved	Follow-Up	Outcomes
Ramanauskaite et al. 2024 [32]	Randomized, controlled, parallel study	48	Generalized Periodontitis (stages II–III)	NR	High-molecular-weight HA	Subgingival debridement	PPD, CAL, BOP, PI	6 months	The PPD and CAL were statistically significant in the test group (*p* < 0.001).
Vela et al. 2024 [33]	Randomized, double-arm, multicentric clinical trial	100	Periodontitis (stages III and IV)	NR	HA gel NR	Debridement only	CAL, PPD, GR	12 months	PPD and CAL were significant at 12 months (*p* < 0.0001).
Axe et al. 2024 [34]	Randomized, clinical study	110	Moderate gingivitis	Modified Gingival Index	High-molecular-weight HA	Fluoride toothpaste	GI, BOP; PI	1, 2 and 6 weeks	No difference was observed between the toothpaste with HA and the control toothpaste (without HA). Both reduced gingival bleeding.
Bertl et al. 2024 [35]	Randomized controlled clinical trial	56	Chronic periodontitis patients (stage III and IV)	World Workshop classification (2017)	HA gel 0.3%	Placebo	CAL, PPD, PI	12 months	Supragingival and subgingival HA gel resulted in fewer sites requiring further intervention.
Benyei et al. 2024 [36]	Pilot randomized controlled clinical trial	52	Residual periodontal pockets	NR	HA gel NR	SRP	BOP, CAL, PPD	3 and 9 months	HA in the experimental group showed an improvement in CAL (*p* = 0.001) and a significant reduction in PPD (*p* = 0.001).
Devina et al. 2024 [37]	Double-blind randomized clinical trial	32	Periodontitis. NR stage	European Federation of Periodontology criteria	HA gel 0.2%	Placebo	BOP, CAL, PPD	4 weeks	The groups showed significant reductions in all clinical parameters (*p* ≤ 0.05), except for PPD and CAL in the placebo group.
Mohammad et al. 2023 [38]	Clinical comparative study	75	Periodontitis. NR stage	NR	HA gel 0.8%	SRP	PI, GI, BOP, PPD, CAL	2 months	Clinical parameters decreased significantly in the experimental group (*p* ≤ 0.001).
Ramanauskaite et al. 2023 [39]	Randomized controlled clinical trial	48	Periodontitis (stages II–III)	NR	HA gel NR	SRP	BOP, PI, PPD, CAL	3 and 6 months	Significant reduction in PPD and BOP in the test group compared to the control group (*p* < 0.001).
Pilloni et al. 2023 [40]	Randomized, split-mouth, single-blind, clinical trial	50	Residual periodontal pockets	NR	HA gel NR	SRP	BOP, PI, PPD, CAL	6, 8, 24, 36, and 48 weeks	The test sites showed a higher percentage of pockets with a PPD ≤ 4 mm. Bleeding decreased in both groups. At sites with baseline PPD values ≥ 6 mm, statistically significant differences were observed between the groups (*p* = 0.004).
Ariel et al. 2022 [41]	Randomized prospective clinical study	34	Periodontitis (stage III)	World Workshop classification (2017)	HA gel NR	SRP	PI, BOP, PPD, CAL	3 and 6 months	PI, PPD, and CAL scores at baseline and follow-up showed a significant reduction in both the test and control groups (*p* < 0.01 for PI and *p* < 0.0001 for PPD and CAL). BOP values were reduced in the test group (*p* < 0.001).
Mamajiwala et al. 2021 [42]	Randomized controlled clinical trial	20	Chronic periodontitis (stage II or III)	Classification of periodontal diseases (1999)	HA gel 0.8%	Debridement + placebo	PI, PPD, CAL, GI, GR	12 months	The test group showed a significantly greater gain in CAL compared to the control group (*p* < 0.001). PPD was significantly reduced in the test group (*p* < 0.05).
Olszewska-Czyz et al. 2021 [43]	Randomized, Controlled Clinical Trial	100	Moderate periodontitis. NR stage	Clinical and radiological examination. 2017 World Workshop on the Classification of Periodontal and Peri-Implant Disease (2017)	HA gel NR	Debridement only	CAL, PPD, BOP	12 weeks	Significant differences between the groups in terms of BOP and CAL in favor of the HA group
Aydinyurt et al. 2020 [44]	Randomized controlled trial	96	Periodontitis. NR stage	NR	HA gel NR	SRP + placebo	BOP, GI, PPD, CAL	4 weeks	BOP, CAL, and PPD were significantly reduced in the HA group (*p* < 0.05).
Al-Shammari et al. 2018 [45]		24	Moderate and Severe Chronic Periodontitis	Classification of periodontal diseases (1999)	HA gel 0.8%	SRP	PI, GI, CAL, PPD	6 and 12 weeks	Statistically significant differences in PPD between the control and test groups (*p* = 0.041 and *p* = 0.02, respectively).
Mallikarjun et al. 2016 [46]	Randomized split-mouth study	20	Chronic periodontitis	NR	HA gel 0.2%	Mechanical debridement	PI, GI, PPD, CAL	6 weeks	The difference in PI, GI, PPD and CAL scores of the control and experimental groups was statistically highly significant (*p* < 0.001).
Eick et al. 2016 [47]	Prospective Randomized Clinical Trial	42	Chronic periodontitis	NR	HA gel 0.8%	SRP	PPD, CAL, BOP, PI	3 and 6 months	The number of sites with PPD ≥ 5 mm decreased more in the test group than in the control group. No differences were observed in CAL, BOP, and PI.
Fawzy El-Sayed et al. 2012 [48]	Randomized controlled trial	28	Chronic periodontitis	NR	HA gel NR	Gel placebo	CAL, BOP, PPD, GR, PI	3 and 6 months	Statistically significant differences in CAL (*p* < 0.05) between the test and control areas.
Johannsen et al. 2009 [49]	Split mouth	12	Chronic periodontitis	NR	HA gel NR	SRP	BOP, CAL, PPD, PI	12 weeks	Significant reduction in PI in the test (*p* < 0.01) and control (*p* < 0.01) groups. PPD was also significantly reduced in the test group (*p* < 0.05).

HA, Hyaluronic acid; SRP, Scaling and root planning; PPD, Pocket probing depth; BOP, Bleeding on probing; CAL, Clinical attachment level; PI, Plaque index; GR, Gingival recession; GI, Gingival index; NR, Not reported.

**Table 4:** Results at 12 months of follow-up for CAL and PPD from the included studies (stage III and IV periodontitis).

Study	Follow-Up	Stage of Periodontitis	Results for CAL Gain	Results for PPD Reduction
Vela et al. 2024 [33]	12 months	Stages III and IV	3.06 ± 1.13 mm (test group) vs. 1.44 ± 1.07 mm (control group); *p* < 0.001 *	3.28 ± 1.14 mm (test group) vs. 2.61 ± 1.22 mm (control group); *p* = 0.032 *
Bertl et al. 2024 [35]	12 months	Stages III and IV	NR	4.2 ± 0.9 mm (test group) vs. 4.5 ± 0.8 mm (control group); *p* = 0.007
Pilloni et al. 2023 [40]	12 months	Residual periodontal pockets after treatment of aggressive periodontitis	(test: −0.50 ± 1.85 mm vs. control: −0.36 ± 1.80 mm). CAL gain was comparable between groups	−2.08 ± 1.24 mm ± 1.24 (test group) vs. −1.94 ± 1.19 ( control group); *p* < 0.0001 *
Mamajiwala et al. 2021 [42]	12 months	Stage III	4.0 ± 0.56 mm (test group) vs. 5.4 ± 0.82 mm (control group); *p* < 0.001 *	3.1 ± 0.58 mm (test group) vs. 4.3 ± 0.47 mm (control group); *p* < 0.0018 *

CAL, Clinical attachment level; PPD, Pocket probing depth; NR, Not reported; * Statistical significance.

**Table 5:** Specific and sociodemographic characteristics of the included studies.

Study	Country	Journal	Age Range	Sex	Tobacco Smokers	Study Registration	Statistical Tests	Dropouts	Adverse Events
Ramanauskaite et al. 2024 [32]	Lithuanian	Oral Health Prev Dent	30 to 72 years	NR	Non-smokers	ClinicalTrials.gov, NCT04662216.	Mann–Whitney, McNemar, Wilcoxon	No dropouts	NR
Vela et al. 2024 [33]	Romania	Medicina (Kaunas)	30 to 60 years	26 females and 34 males	Non-smokers	Clinical Trials (NCT05073575)	Kolmogorov–Smirnov, Chi-square, Fisher’s	No dropouts	NR
Axe et al. 2024 [34]	Canada	BMC Oral Health	18 to 65 years	66 females and 44 males	Non-smokers	International Council for Harmonization of Technical Requirements for Registration of Pharmaceuticals for Human Use Good Clinical Practice	ANCOVA	No dropouts	Reported adverse events
Bertl et al. 2024 [35]	Sweden	Clin Oral Investig	35 to 75 years	20 females and 36 males	Non-smokers	Clinicaltrials.gov (NCT04792541)	Chi-squared, Mann–Whitney-U, Shapiro–Wilk	No dropouts	No Adverse events
Benyei et al. 2024 [36]	Germany	Clin Oral Investig	49.3 ± 11.2 47.3 ± 10.7	35 females and 13 males	Non-smokers	ClinicalTrials.gov, NCT04662216	Shapiro–Wilk, Wilcoxon, Mann–Whitney	No dropouts	No Adverse events
Devina et al. 2024 [37]	Indonesia	Eur J Dent	31–71 years	24 females and 26 males	9 (≤10 cigarettes/day)	(ClinicalTrials.gov- NCT05210686)	Shapiro–Wilk	No dropouts	No Adverse events
Mohammad et al. 2023 [38]	Irak	Gels	29–78 years	21 males and 13 females	Smoking less than 10 cigarettes/day	Israeli Ministry of Health (0034–17-MHMC).	NR	No dropouts	Reported adverse events
Ramanauskaite et al. 2023 [39]	Lithuanian	Clin Oral Investig	34 to 51 years	11 males and 9 females	Non-smokers	Institutional Ethical Committee Maharashtra University of Health Sciences, Nashik (MGV/KBHC/786/2016-17)	Shapiro–Wilk, Student’s t, Mantel–Haenszel χ^2^	No dropouts	NR
Pilloni et al. 2023 [40]	Italy	J Periodontol	25 to 65 years	51% women	Non-smokers (for a minimum of 5 years)	Jagiellonian University Ethics Committee (122.6120.132.2015)	Mann–Whitney U, Shapiro–Wilk	No dropouts.	No Adverse events
Ariel et al. 2022 [41]	Israel	Clin Oral Investig	18 to 55 years	40 males, 56 females	Non-smokers	ClinicalTrials.gov.tr (NCT03754010).	Kruskal–Wallis, Turkey, Kramer, Bonferroni	No dropouts	NR
Mamajiwala et al. 2021 [42]	India	Clin Oral Investig	24 to 57 years	14 females, 10 males	Non-smokers	Riyadh Elm University. (RC/IRB/2016/478)	Shapiro, Mann–Whitney U, Wilcoxon	2 dropped out	
Olszewska-Czyz et al. 2021 [43]	Croatia	Biomolecules	20–60 years	11 males and nine females	Non-smokers	Institutional Ethics Committee, Department of Periodontics, Dayananda Sagar College of Dental Sciences, Bengaluru, India	Student’s t, Pearson’s correlation	No dropouts	NR
Aydinyurt et al. 2020 [44]	Turkey	Irish Journal of Medical Science.	41 to 72 years	18 males and 24 females		Ethics Commission (#121 in 2006) of the University of Leipzig Medical Faculty	Wilcoxon, U-test	8 dropped out	No Adverse events
Al-Shammari et al. 2018 [45]	Saudi Arabia	J Contemp Dent Pract.	NR	NR	Non-smokers	Ethical Committee at Cairo University Hospital, Cairo, Egypt.	Shapiro–Wilk, Wilcoxon, McNemar, Friedman, Cochran	2 dropped out	NR
Mallikarjun et al. 2016 [46]	India	Indian J Dent Res.	42 to 63 years	7 males and 5 women	Two were smokers, and one used snuff	Ethics Committee at Huddinge University Hospital, Huddinge, Sweden	Wilcoxon	No dropouts	NR
Eick et al. 2016 [47]	Switzerland	J Periodontol	41 to 72 years	18 males and 24 females		Ethics Commission (#121 in 2006) of the University of Leipzig Medical Faculty	Wilcoxon, U-test	8 dropped out	No Adverse events
Fawzy El-Sayed et al. 2012 [48]	Egypt	Clin Oral Investig.	NR	NR	Non-smokers	Ethical Committee at Cairo University Hospital, Cairo, Egypt.	Shapiro–Wilk, Wilcoxon, McNemar, Friedman, Cochran	No dropouts	NR
Johannsen et al. 2009 [49]	Sweden	J Periodontol.	42 to 63 years	7 males and 5 women	Two were smokers, and one used snuff	Ethics Committee at Huddinge University Hospital, Huddinge, Sweden	Wilcoxon	No dropouts	NR

## Data Availability

All data are available in the manuscript.

## Supplementary Materials

The following supporting information can be downloaded at: https://www.mdpi.com/article/10.3390/bioengineering12111135/s1, Table S1. PRISMA Checklist.

## Abbreviations

The following abbreviations are used in this manuscript:

HA	hyaluronic acid
PPD	pocket probing depth
BOP	bleeding on probing
CAL	clinical attachment level
PI	plaque index
GI	gingival index
SMDs	standardized mean differences
CI	confidence intervals
RCTs	randomized clinical trials
MeSH	Medical Subject Headings
JBI-MAStARI	Joanna Briggs Institute Meta-Analysis of Statistics Assessment and Review Instrument
GR	gingival recession
