# Supplementary material for: The Role of Hyaluronic Acid in the Treatment of Gingivitis and Periodontitis at Different Stages: A Systematic Review and Meta-Analysis with Short-Term Follow-Up
Source: Bioengineering (Basel). 2025 Oct 22;12(11):1135. doi: 10.3390/bioengineering12111135 (PMC12649663; doi:10.3390/bioengineering12111135)
Supplement: Supplementary file 1 [file bioengineering-12-01135-s001.zip › bioengineering-3875869-supplementary.pdf]

PRISMA 2020 Checklist

Table S1. PRISMA Checklist.

Section and Topic	Item #	Checklist item	Location where item is reported
**TITLE**			
Title	1	Role of hyaluronic acid in periodontal treatment: A systematic review and meta-analysis	1
**ABSTRACT**			
Abstract	2	Periodontal diseases are inflammatory conditions that destroy the periodontal attachment apparatus. Hyaluronic acid (HA) has anti-inflammatory properties that make it a candidate for the adjuvant treatment of gingivitis and periodontitis. Our objective was to evaluate the role of HA in the treatment of these conditions.<br>This systematic review and meta-analysis was conducted according to Cochrane guidelines, and searches were performed in PubMed, Embase, Cochrane Central, Scopus, and Web of Science (WOS) to identify eligible studies. Review Manager 5.4.1 and SPSS Statistics 30.0® were used to calculate standardized mean differences (SMDs) and 95% confidence intervals (CIs). The outcomes assessed were probing depth (PPD), bleeding on probing (BOP), clinical attachment level (CAL), plaque index (PI), and gingival index (GI).<br>Sixteen randomized clinical trials (RCTs) with 947 subjects were included. AH as an adjunct to periodontal treatment improves the clinical parameters of PPD in the short and medium term, BOP, CAL, and GI. Plaque indices (PI) approached statistical significance.<br>Despite limitations and heterogeneity, the evidence supports the short- and me-dium-term benefits of applying AH as an adjunct to periodontitis treatment.	2
**INTRODUCTION**			
Rationale	3	In patients with gingivitis and periodontitis, HA has been used as an adjuvant therapy for scaling and root planning therapy (SRP), having demonstrated its ability to reduce prostaglandins, metalloproteinases and bioactive materials, which would hinder tissue destruction, favoring healing [17]. Other studies have also demonstrated its use-fulness in gingivitis, in topical application, decreasing bleeding and improving gingival health [18,19] (Figure 1). There are few systematic reviews [20-24] recommended every 2 to 5 years [25], so the aim of our study was to evaluate the clinical efficacy of AH in gingivitis and periodontitis through a systematic review and meta-analysis.	2
Objectives	4	The aim of our study was to evaluate the clinical efficacy of AH in gingivitis and periodontitis through a systematic review and meta-analysis.	2
**METHODS**			
Eligibility criteria	5	The research studies were selected according to the following inclusion criteria: (i) randomized clinical trials (single or double blind) that included in the study more than 10 adult subjects (≥ 18 years of age); (ii) suffering from gingivitis or periodontitis; (iii) that provided data on clinical parameters indicative of these pathologies; (iv) with statistical methods that included means and standard deviation, together with units with which to quantify mediator levels; (v) published in English. Studies that did not follow all the criteria defined above, with lack of data demonstrating periodontal disease, in vitro or experimental animal studies, case series or clinical cases, literature reviews and irrelevant studies, such as editorials, contributions to congresses, etc., were excluded.	3
Information sources	6	Table 1	3
Search strategy	7	Table 2	4
Selection process	8	Table 2	5
Data collection process	9	Two reviewers (NL-V and AL-V) extracted and tabulated the data from each included study using the standardized data extraction tool "The Joanna Briggs Institute Me-ta-Analysis of Statistics Assessment and Review Instrument" (JBI-MAStARI) [26]. The two reviewers reviewed the titles and abstracts of the pre-selected studies. Those that met the inclusion criteria were read in full and the data extracted. Disagreements between the reviewers were resolved through discussion and mediation by a third reviewer (JABR). Cohen's kappa (κ) index [27] was used to assess inter-rater agreement. The data extracted from the studies included specific details of the interventions, study methods, populations, specific objectives and significant results to formulate the question of interest. The results were entered twice to minimize error bias.	4
Data items	10a	List and define all outcomes for which data were sought. Specify whether all results that were compatible with each outcome domain in each	----

PRISMA 2020 Checklist

Section and Topic	Item #	Checklist item	Location where item is reported
		study were sought (e.g. for all measures, time points, analyses), and if not, the methods used to decide which results to collect.	
	10b	List and define all other variables for which data were sought (e.g. participant and intervention characteristics, funding sources). Describe any assumptions made about any missing or unclear information.	----
Study risk of bias assessment	11	GRADE criteria	5
Effect measures	12	Specify for each outcome the effect measure(s) (e.g. risk ratio, mean difference) used in the synthesis or presentation of results.	----
Synthesis methods	13a	Describe the processes used to decide which studies were eligible for each synthesis (e.g. tabulating the study intervention characteristics and comparing against the planned groups for each synthesis (item #5)).	----
	13b	Describe any methods required to prepare the data for presentation or synthesis, such as handling of missing summary statistics, or data conversions.	----
	13c	Describe any methods used to tabulate or visually display results of individual studies and syntheses.	----
	13d	Describe any methods used to synthesize results and provide a rationale for the choice(s). If meta-analysis was performed, describe the model(s), method(s) to identify the presence and extent of statistical heterogeneity, and software package(s) used.	----
	13e	Describe any methods used to explore possible causes of heterogeneity among study results (e.g. subgroup analysis, meta-regression).	----
	13f	Describe any sensitivity analyses conducted to assess robustness of the synthesized results.	----
Reporting bias assessment	14	Describe any methods used to assess risk of bias due to missing results in a synthesis (arising from reporting biases).	----
Certainty assessment	15	Describe any methods used to assess certainty (or confidence) in the body of evidence for an outcome.	----
**RESULTS**			
Study selection	16a	Of the 1,563 records identified, 1,285 were eliminated (duplicates, case reports, preclinical studies, literature reviews); subsequently, 234 were eliminated (text not accessible, lack of relevance, and communication of other results); finally, of the 44 studies selected for evaluation, 26 were eliminated for various reasons, leaving 18 studies for the systematic review and only 16 studies for meta-analysis]. The agreement between reviewers when including studies exceeded 85% (κ > 85%).	6
	16b		----
Study characteristics	17	Tables 3 and 4	8-14
Risk of bias in studies	18	Table 5	17
Results of individual studies	19	For all outcomes, present, for each study: (a) summary statistics for each group (where appropriate) and (b) an effect estimate and its precision (e.g. confidence/credible interval), ideally using structured tables or plots.	----
Results of syntheses	20a	For each synthesis, briefly summarise the characteristics and risk of bias among contributing studies.	----
	20b	Present results of all statistical syntheses conducted. If meta-analysis was done, present for each the summary estimate and its precision (e.g. confidence/credible interval) and measures of statistical heterogeneity. If comparing groups, describe the direction of the effect.	----
	20c	Present results of all investigations of possible causes of heterogeneity among study results.	----
	20d	Present results of all sensitivity analyses conducted to assess the robustness of the synthesized results.	----
Reporting biases	21	Present assessments of risk of bias due to missing results (arising from reporting biases) for each synthesis assessed.	----

PRISMA 2020 Checklist

Section and Topic	Item #	Checklist item	Location where item is reported
Certainty of evidence	22	Present assessments of certainty (or confidence) in the body of evidence for each outcome assessed.	----
**DISCUSSION**			
Discussion	23a	We found that all included meta-analyses re-ported benefits of periodontal treatment on the periodontal parameters studied (PPD, CAL, BOP, GI, PL) and some cytokines (IL-1β and TNF-α).	18
	23b	Limitations. The studies included in our meta-analysis showed a high publication bias, in ad-dition to considerable heterogeneity, which was between 79 and 90%, something that could be influenced by differences in the severity of the disease (different stages), dif-ferences between the populations studied, formulations and application of HA and lo-cation of the defects, which makes it difficult to evaluate the test product. In addition, the follow-up times and the different statistical programs used in the different studies, can lead to biases in the communication and interpretation of the results. The inclusion of publications exclusively in English and databases that were not consulted could also lead to publication bias. Finally, it is worth mentioning an aspect, referring to the Hawthorne effect [65], which causes changes in the behavior of people who feel observed in epidemiological studies, something that was not taken into account in any of the included studies.	21
	23c	Discuss any limitations of the review processes used.	----
	23d	Discuss implications of the results for practice, policy, and future research.	----
**OTHER INFORMATION**			
Registration and protocol	24a	The protocol of this meta-analysis has been registered in PROSPERO ID: CRD42024626469 in date 22/12/2024.	3
	24b	Indicate where the review protocol can be accessed, or state that a protocol was not prepared.	----
	24c	Describe and explain any amendments to information provided at registration or in the protocol.	----
Support	25	Non-financial support for the review	21
Competing interests	26	The authors declare no conflicts of interest	21
Availability of data, code and other materials	27	Report which of the following are publicly available and where they can be found: template data collection forms; data extracted from included studies; data used for all analyses; analytic code; any other materials used in the review.	----
